# A Cylindrical Molding Method for the Biofabrication of Plane-Shaped Skeletal Muscle Tissue

**DOI:** 10.3390/mi12111411

**Published:** 2021-11-17

**Authors:** Minghao Nie, Ai Shima, Kenta Fukushima, Yuya Morimoto, Shoji Takeuchi

**Affiliations:** 1 Department of Mechano-Informatics, Graduate School of Information Science and Technology, The University of Tokyo, 7-3-1 Hongo, Bunkyo-ku, Tokyo 113-8656, Japan; nie@hybrid.t.u-tokyo.ac.jp (M.N.); shima@hybrid.t.u-tokyo.ac.jp (A.S.); y-morimo@hybrid.t.u-tokyo.ac.jp (Y.M.); 2 Department of Mechano-Informatics, Faculty of Engineering, The University of Tokyo, 7-3-1 Hongo, Bunkyo-ku, Tokyo 113-8656, Japan; kenta_fukushima@hybrid.t.u-tokyo.ac.jp; 3 Institute of Industrial Science (IIS), The University of Tokyo, 4-6-1 Komaba, Meguro-ku, Tokyo 153-8505, Japan; 4 International Research Center for Neurointelligence (WPI-IRCN), The University of Tokyo Institutes for Advanced Study, The University of Tokyo, 7-3-1 Hongo, Bunkyo-ku, Tokyo 113-8656, Japan

**Keywords:** biofabrication, tissue engineering, skeletal muscle, microtissue assembly

## Abstract

Muscle tissues can be fabricated in vitro by culturing myoblast-populated hydrogels. To counter the shrinkage of the myoblast-populated hydrogels during culture, a pair of anchors are generally utilized to fix the two ends of the hydrogel. Here, we propose an alternative method to counter the shrinkage of the hydrogel and fabricate plane-shaped skeletal muscle tissues. The method forms myoblast-populated hydrogel in a cylindrical cavity with a central pillar, which can prevent tissue shrinkage along the circumferential direction. By eliminating the usages of the anchor pairs, our proposed method can produce plane-shaped skeletal muscle tissues with uniform width and thickness. In experiments, we demonstrate the fabrication of plane-shaped (length: ca. 10 mm, width: 5~15 mm) skeletal muscle tissue with submillimeter thickness. The tissues have uniform shapes and are populated with differentiated muscle cells stained positive for myogenic differentiation markers (i.e., myosin heavy chains). In addition, we show the assembly of subcentimeter-order tissue blocks by stacking the plane-shaped skeletal muscle tissues. The proposed method can be further optimized and scaled up to produce cultured animal products such as cultured meat.

## 1. Introduction

The biofabrication of muscle tissue aims at the in vitro reconstruction of muscle tissues, using cells and biomaterials to replicate the compositions, morphologies, and functions of in vivo muscle tissues, which has broad applications in drug development [1,2,3], biohybrid robotics [4,5,6,7,8], regenerative medicine [9,10,11], and cellular agriculture [12,13,14]. The general biofabrication process of muscle tissue starts from the crosslinking of a hydrogel matrix populated with myoblasts, since some types of cells (such as fibroblasts and myoblasts) can adhere to the matrix, exerting mechanical forces to and remodeling the matrix [15]. Furthermore, to encourage the migration and fusion of myoblasts, low-concentration hydrogels are preferred to dense polymer networks which are often used for bioprinting purposes [16,17] and can lead to drastic shrinking within the culture due to the lack of mechanical stiffness. To prevent the over-shrinkage of the myoblast-populated hydrogel matrix, anchoring methods are proposed to reinforce the tissue by fixing the two ends of the myoblast-populated hydrogel using a pair of anchors/pillars [18,19,20]. The anchors can not only prevent the drastic shrinkage of the myoblast-populated hydrogel but can also sustain the tensions generated within the tissue to promote the differentiation of myoblasts and mature the muscle tissues. In addition, the fabricated muscle tissues can be further assembled using methods such as stacking to create macro-scale tissues [13]. However, the anchoring methods cannot effectively fabricate a muscle tissue with uniform width and thickness, as the tissues far from the anchors often shrink more than the tissues surrounding the anchors; uniform width and thickness are necessary for assembling the tissues seamlessly into macro-scale tissue.

**Figure 1:**
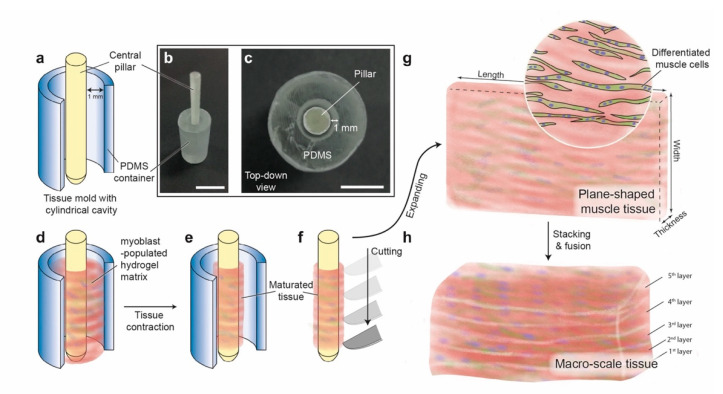
Concept of the cylindrical molding method for the biofabrication of plane-shaped skeletal muscle tissue. (**a**) Design of the tissue mold with a cylindrical cavity consisting of a 3D-printed central pillar and a cylindrical container made of polydimethylsiloxane (PDMS) for better oxygen permeability. (**b**) Photo image of the tissue mold (perspective view). (**c**) Photo image of the tissue mold (top-down view). (**d**,**e**) The cylindrical molding method starts by casting myoblast-populated hydrogel matrix into the cylindrical cavity of the tissue mold, followed by subsequent maturation of the culture during which the tissue shrinks and the cells differentiate. (**f**) The maturated tissue is collected by withdrawing the central pillar and is subsequently cut vertically. (**g**) Plane-shaped muscle tissue is acquired after expanding the cut tissue. (**h**) A macro-scale tissue block can be constructed by stacking multiple pieces of plane-shaped muscle tissues. Scale bars: (**b**) 1 cm; (**c**) 5 mm.

In this work, we propose a cylindrical molding method for the formation of plane-shaped muscle tissue, i.e., hydrogel sheets consisting of differentiated skeletal myoblasts. As shown in Figure 1, after molding the myoblast-populated hydrogel matrix in a cylinder cavity with a central pillar (referred to as a ‘tissue mold’), the shrinkage of the tissue is constrained by the central pillar. Therefore, plane-shaped skeletal muscle tissues with uniform width and thickness can be produced without the usages of multiple anchors. In experiments, we demonstrate the fabrication of plane-shaped (length: ca. 10 mm, width: 5~15 mm) skeletal muscle tissue with submillimeter thickness. The tissues have uniform shapes and are populated with differentiated myoblasts stained positive for myogenic differentiation markers (i.e., myosin heavy chains). In addition, we show the assembly of subcentimeter-order tissue blocks by stacking the plane-shaped skeletal muscle tissues.

## 2. Materials and Methods

### 2.1. Design and Fabrication of the Tissue Mold

The tissue mold used for the cylindrical molding method consists of two parts: a central pillar and a cylindrical polydimethylsiloxane (PDMS) container (Figure 1a). The central pillar is a cylinder (diameter: 3.2 mm, height: 30 mm) with its bottom connected to a cone extended downward for 5 mm. The target length of the tissue is 1 cm, which correlates to ca. 3.2 mm diameter of the central pillar. The cone-shaped bottom is for effective plugging of the pillar into the cylindrical container. The cylindrical PDMS container has a cylindrical cavity (diameter: 5.2 mm, depth: 20 mm), and an additional cone-shaped cavity is beneath the cylindrical cavity with the same geometry as the bottom cone of the central pillar to ensure stable and vertical plug-in of the central pillar. After plugging the central pillar, the overlapping length of the central pillar and the cylindrical cavity is 20 mm, which establishes the initial height of the molded tissue with a maximum chamber volume of ca. 0.2 mL (when plugged with the central pillar). A 3D printer (Agilista-3200, Keyence, Osaka, Japan) was used to fabricate the central pillar and the mold for fabricating the cylindrical PDMS container. After printing, all parts were thoroughly washed using water to remove the supporting materials, dried overnight, and coated with parylene-C using a parylene coater (Parylene Deposition System 2010, Specialty Coating Systems, Indianapolis, IN, USA). To fabricate the cylindrical PDMS container, PDMS precursors (two-component, mixed at a 10:1 ratio, SYLGARD™184 Silicone Elastomer Kit, The Dow Chemical Company, Midland, MI, USA) were poured into the parylene-coated mold, subsequently heated at 75 °C for 90 min, and demolded after PDMS crosslinking.

### 2.2. Cell Culture and Tissue Construction

Mouse C2C12 myoblasts were expanded in Dulbecco’s Modified Eagle Medium (DMEM) (high-glucose) (Nacalai Tesque, Kyoto, Japan) containing 10% fetal bovine serum (Thermo Fisher Scientific, Waltham, MA, USA) and penicillin-streptomycin (Merck, Darmstadt, Germany) (growth medium (GM)) at 37 °C in 5% CO_2_. For constructing the plane-shaped skeletal muscle tissue, C2C12 cells were mixed in native collagen from the bovine dermis, AteloCell IAC-50 (5 mg/mL) (KOKEN, Tokyo, Japan), or AteloCell IAC-50 mixed with an equal amount of Matrigel (Corning, Glendale, AZ, USA) at 2.5 × 10^7^ cells/mL. AteloCell IAC-50 was neutralized by 10× phosphate-buffered saline (PBS)(-) or a mixture of 10× Hank’s Balanced Salt Solution, HEPES (pH 7.4), NaHCO_3_, and H_2_O. After being poured into the cylindrical tissue mold, the hydrogel containing the cells was gelled by placing in an incubator (37 °C, humidified) for 30 min. After gelation, the tissues (with the central pillar) were plugged into a PDMS culture chamber for subsequent culture. The PDMS culture chamber has the same shape as the cylindrical PDMS container for tissue molding (described in Section 2.1) except for a larger cavity volume to contain culture medium (10 mL). Then, GM was added to the large PDMS containers, and the tissues were cultured for 24 h. Next, the medium was replaced with DMEM (low-glucose) (Fujifilm Wako Pure Chemical, Osaka, Japan) containing 10% horse serum (Thermo Fisher Scientific, Waltham, MA, USA) and penicillin-streptomycin (differentiation medium (DM)) on Day 1, and the tissues were cultured in DM for 6 more days at 37 °C in 5% CO_2_. The medium was fully exchanged every other day. On Day 7, the tissue was taken out from the medium and cut into sheets by scalpel.

### 2.3. Cell Distribution Analysis

Cell nuclei in the plane-shaped skeletal muscle tissue were stained with Hoechst 33342 (Thermo Fisher Scientific, Waltham, MA, USA) and the images were taken by a confocal laser scanning microscopy LSM780 (Carl Zeiss, Oberkochen, Germany). The number of cell nuclei per unit area was counted using ImageJ (National Institutes of Health, Bethesda, MD, USA). In detail, images were binarized with thresholds (double-checked by comparing the images before and after thresholding), processed using the watershed segmentation algorithm, and counted using the Analyze Particle function.

### 2.4. Fluorescent Staining of Plane-Shaped Skeletal Muscle Tissue

For immunofluorescence, the tissues were fixed with 4% paraformaldehyde (PFA) for 30 min, treated in 0.1% Triton X-100 for 30 min for cell permeabilization, and incubated in 1% bovine serum albumin in PBS(-) for blocking. MF20 (Developmental Studies Hybridoma Bank, Iowa City, IA, USA) was used as a primary antibody to detect myosin heavy chains, and Alexa Fluor 488 goat anti-mouse IgG (Thermo Fisher Scientific, Waltham, MA, USA) was used as a secondary antibody. Cell nuclei were stained with Hoechst 33342. The images were taken by the LSM780. To evaluate the myogenic differentiation, the number of myotubes with 2 or more nuclei per unit area was counted using ImageJ.

For filamentous actin staining, the tissue was fixed and treated in Triton X-100 as mentioned above and incubated with Alexa Fluor 488-conjugated Phalloidin (Thermo Fisher Scientific, Waltham, MA, USA) for 1 h. The image of whole tissue was taken by THUNDER Imaging systems (Leica microsystems, Wetzlar, Germany).

### 2.5. Width and Thickness Measurement of the Tissue

To measure the width of the tissue, images of the cut and expanded tissues were taken from a top-down angle with rulers in the frame so that image pixel distances can be converted to standard measurement units. Widths of the tissues were measured using ImageJ.

To measure the thickness of the tissue (for both a single tissue and the stacked tissues), we prepared the setup consisting of a modified Petri dish (Figure S1) and a microscope (VW9000, Keyence). The Petri dish was cut straightly into two pieces (a larger piece and a smaller piece). The larger piece was glued with a piece of Poly (methyl methacrylate) (PMMA) slab so that the sidewall of the Petri dish was perfectly flat and transparent. By placing the microscope horizontally, images of the tissue (placed on another PMMA slab placed inside the Petri dish) can be taken through the PMMA slab to avoid light refraction. The tissues were submerged in PBS(-) and the liquid level was lowered beneath the upper surface of the PMMA slab (on top of which the tissues were placed) only during imaging. The thickness was measured from the images by using ImageJ.

### 2.6. Tissue Block Assembly by Stacking the Plane-Shaped Skeletal Muscle Tissues

For stacking the plane-shaped skeletal muscle tissues, we used a jig with walls to assist the placement of tissues, which was made by a 3D printer (Agilista-3200) and coated with parylene-C (Figure S2, upper). As an example of stacking two layers of tissues (Figure S2, lower), the plane-shaped tissues and a jig were first placed in the Petri dish (modified for thickness measurement according to Section 2.5) and the liquid level of PBS(-)/medium was adjusted to submerge the base of the jig with an additional height approximately the same as the thickness of a piece of the plane-shaped tissue (otherwise, the tissue would float in the liquid after being placed on the jig). After placing the tissue, the liquid level was lowered beneath the bottom of the tissue to allow imaging of the tissue (as described in detail in Section 2.5). Then, the liquid level was adjusted to submerge the top surface of the stacked tissues with an additional height approximately the same as the thickness of a piece of the plane-shaped tissue. Next, another tissue was stacked, and the thickness of the overall tissue block was measured with the liquid level lowered beneath the bottom of the tissue block. For stacking more layers of the tissues, the adjustment of the liquid level was performed repetitively.

For the adhesion treatment of the stacked tissues, a small amount of collagen gel (AteloCell IAC-50, 5 mg/mL) was dropped onto the tissue block while the tissues were just piled up as negative control. For convenience, quarter-sized muscle tissues were prepared by cutting each plane-shaped tissue into four pieces. To investigate the necessity and efficiency of the adhesion treatment, two pieces of the quarter-sized muscle tissues were stacked in PBS(-) with or without the adhesion treatment, and then manually shaken for 1 min (frequency ca. 0.5 Hz, amplitude ca. 1 cm), 30 min after stacking, or after overnight incubation in DM.

### 2.7. Statistical Analysis

Three or more samples were measured/counted to collect the data. All data were shown as the mean ± SD. The statistical significance (P ≤ 0.05) was evaluated by Welch’s *t*-test between the two groups (stats.ttest_ind, SciPy, Python) and by TukeyHSD test among the three groups (pairwise_tukeyhsd, statsmodels, Python).

## 3. Results and Discussions

### 3.1. Shape and Homogeneity of the Biofabricated Plane-Shaped Skeletal Muscle Tissues

One of the advantages of using the central pillar to counter the tissue shrinkage is that the length of the fabricated tissue is mainly decided by the peripheral length of the central pillar, since the circumferential shrinkage will force the tissue to firmly grab the central pillar. On the contrary, the tissue can be rather free to shrink along the width directions, and the friction between the tissue and the surface of the central pillar could be the only counterforce to shrinkage. In addition, since the vertical direction also aligns with gravity during the crosslinking of the cell-laden hydrogel precursors, sedimentation of the cells along the vertical direction before the full crosslinking of the hydrogels could lead to the inhomogeneous distribution of cells in the tissue.

**Figure 2:**
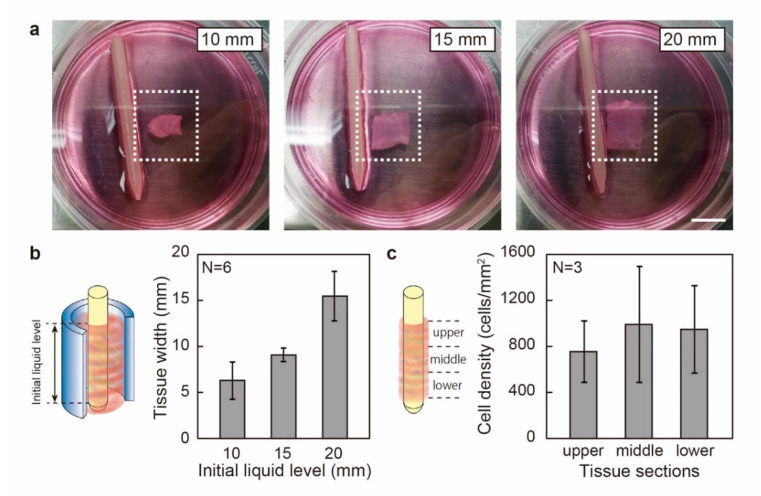
Shape and homogeneity of the biofabricated plane-shaped muscle tissue. (**a**) Images of the tissues fabricated with various initial liquid levels. The tissues are placed so that the length directions are roughly aligned with the horizontal directions. (**b**) The measured widths of the tissues fabricated with the various initial liquid levels. (**c**) Cell densities for the upper, middle, and lower sections of the fabricated tissue. Scale bar: 1 cm.

In experiments, we first measured the shape of the tissue fabricated with different initial liquid levels of the cell-laden hydrogel precursors. Figure 2a shows the images of the cut and expanded tissues with various initial liquid levels. The measured widths of the tissues are 6.3 ± 2.1 mm, 9.0 ± 0.8 mm, and 15.4 ± 2.7 mm for the initial liquid level of 10, 15, and 20 mm, respectively (Figure 2b). The shrinkage percentages of the tissues against initial liquid levels were calculated to be 37%, 40%, and 23%, respectively. The shrinkage percentage after tissue shrinkage does not change linearly with the initial liquid level, while the lowest shrinkage rate of 23% is found for the tissues with an initial liquid level of 20 mm. Then, the tissues fabricated with an initial liquid level of 20 mm were stained with Hoechst 33342, and the cell nuclei were sorted into three separate sections (i.e., upper, middle, and lower) along the vertical direction and counted. As shown in Figure 2c, the cell densities were 755 ± 267 cells/mm^2^, 992 ± 504 cells/mm^2^, and 948 ± 380 cells/mm^2^ for the upper, middle, and lower sections. Despite the upper section resulting in a slightly lower mean value of nuclei numbers, which could be a result of cell sedimentation, differences between the three groups were statistically insignificant (TukeyHSD, P ≤ 0.05, N = 3). It is also worth mentioning that no apparent warpage of tissues was observed after cutting and expanding the tissue, which indicates that the attenuation of internal stress within the tissue was possibly due to the remodeling of the matrix by the C2C12 cells.

### 3.2. Optimization on the Hydrogel Composition to Facilitate Myogenic Differentiation

**Figure 3:**
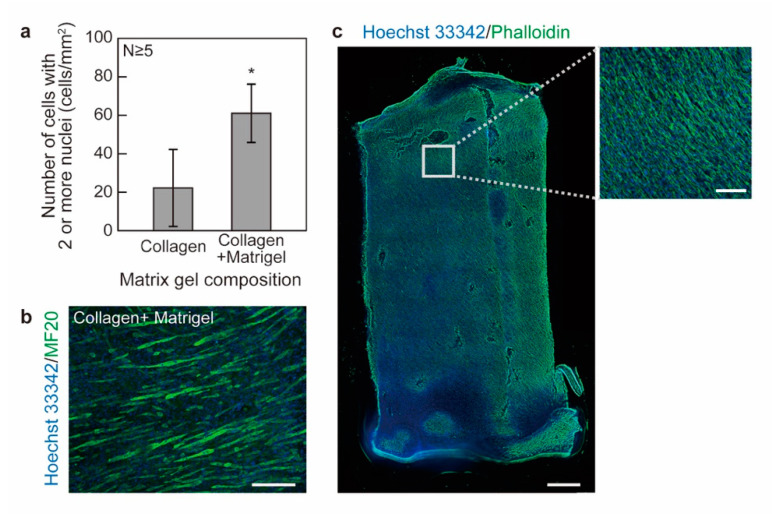
Optimization of the hydrogel composition to facilitate myogenic differentiation. (**a**) The numbers of cells with two or more nuclei per area for the tissues fabricated with/without the addition of Matrigel. * P ≤ 0.05, Welch’s *t*-test (N ≥ 5). (**b**) A representative image of the tissue stained with Hoechst 33342/MF20. (**c**) Tile scan of a whole piece of tissue stained with Hoechst 33342/Phalloidin. The inset shows a magnified image of the tissue in the rectangular region. Scale bars: (**b**) 200 µm, (**c**) 500 µm (inset: 100 µm).

Myogenic differentiation of the myoblasts is essential for the construction of muscle tissue. For C2C12 cells, various factors are reported to affect the myogenic differentiation, such as the composition of hydrogel matrix and the tension generated within the tissues, the use of a basement membrane-like matrix (e.g., Matrigel) as a substrate for 2D cultures, or for 3D cultures showing enhanced myogenic differentiation [21], and the use of anchors (that can maintain the tension generated due to tissue shrinkage) to mimic the in vivo muscle tissue morphology [18,19,20]. In experiments, we focused on the optimization of the hydrogel matrix composition by comparing the difference between the tissues fabricated using a collagen matrix with/without the addition of Matrigel. As a result, the numbers of cells with two or more nuclei were counted per area and plotted, as shown in Figure 3a, which indicated a statistically significant increase in the numbers of cells with two or more nuclei (Welch’s *t*-test, P ≤ 0.05, N ≥ 5). Since the cells with two or more nuclei are the result of myoblast fusion during myogenesis, it can be concluded that the tissues fabricated with the addition of Matrigel are more differentiated than the ones without the addition of Matrigel. A representative MF20 immunostaining image of tissues fabricated with the addition of Matrigel is shown in Figure 3b.

To better inspect the cell distribution within the whole area of the tissue, a tile scan of a tissue stained with Hoechst 33342/Phalloidin was performed with the images merged, as shown in Figure 3c, with the inlet highlighting the confluency of cells within the tissue. The overall length of the tissue was less than 1 cm due to the shrinkage caused by 4% PFA used for tissue fixation. The localized alignment of the cells can be confirmed, as shown in Figure 3c. Though a shift of the cell alignment direction was apparent, there are very few sharp changes in cell alignment direction across the whole sheet. We hypothesize the surface roughness of the central pillar to be one important factor affecting the cell alignment within the whole sheet, since friction between the muscle tissue and the central pillar surface could also counter the shrinkage of the tissue. Optimization of the surface roughness of the central pillar can be performed by polishing the pillar surfaces, or by printing the central pillar using 3D printers with higher levels of precision. Despite some damaged areas, which could be caused by the cutting of the tissue and subsequent tissue handling, a homogeneous cell distribution within the tissue was achieved. Since the tissue has a submillimeter thickness, the methods for assessing cell viability such as the live/dead staining using calcein-AM/ethidium homodimer-1 are challenging; timing control of the staining will be affected by the lag of molecule diffusion through the thick tissue. Instead, we use cell nuclei to evaluate tissue necrosis since the staining agents for cell nuclei, such as DAPI and Hoechst 33342, have small molecular weights and can diffuse quickly within thick tissue. As a result, the positive staining of cell nuclei within the whole sheet, as shown in Figure 3c, indicates no apparent necrosis. Furthermore, the appearance of cells with more than two nuclei could indicate the activity of the cells during the period of differentiation culture. Due to the relatively short differentiation period (7 days), tissue contraction was not detected. Longer periods of differentiation culture (> 10 days) are speculated to be necessary to further achieve the contractile function of the muscle tissue in future works.

### 3.3. Assembly of Subcentimeter-Order Tissue Block by Stacking the Plane-Shaped Skeletal Muscle Tissues

**Figure 4:**
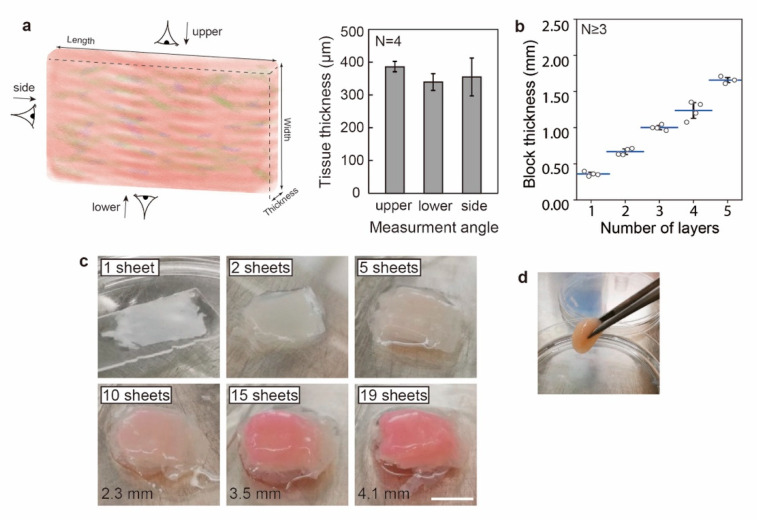
Assembly of subcentimeter-order tissue block by stacking the plane-shaped skeletal muscle tissues. (**a**) Thickness of a single piece of muscle tissue measured in various regions. (**b**) The thickness of the assembled tissue after stacking with various layers of plane-shaped skeletal muscle tissue. (**c**) Photo images of the assembled tissues. (**d**) Photo image of the assembled tissue manipulated by a tweezer. Scale bars: 1 cm.

The thickness of a single piece of the plane-shaped muscle tissue was measured after tissue shrinkage. Due to difficulties in imaging the tissue when mounted on the central pillar, the tissues were assessed after being cut and expanded, with their thicknesses measured from three regions (upper, lower, and side). As shown in Figure 4a, the thicknesses of the plane-shaped skeletal muscle tissues were 385 ± 16 µm, 339 ± 25 µm, and 354 ± 58 µm for the upper, lower, and side regions of the tissues, respectively. Differences between the three groups were statistically insignificant (TukeyHSD, P ≤ 0.05, N = 4). Since the initial thickness of the tissue was designed to be 1 mm, the tissues shrank to ca. 40% of their initial size along the radial direction during the maturation period.

Subcentimeter-order tissue blocks were assembled by stacking the plane-shaped muscle tissues. To enhance the adhesion between the sheets, collagen adhesion treatments were performed during the stacking process. To be precise, a small amount of collagen solution was applied to the tissue block immediately after stacking so that the subsequent crosslinking of the collagen gel can glue the sheets together. To test the effectiveness of the adhesion treatment, shaking tests were performed for the samples (stacked using two pieces of tissue) with/without the application of the collagen solution after two intervals of 30 min and overnight. With the shaking motion applied to the culture dishes, 6/6 of the tissues with the collagen adhesion treatment remained integrated. On the contrary, for the tissues without the collagen adhesion treatment, 3/6 of the tissues were shaken apart 30 min after the stacking; with longer incubation time (overnight), the integrity of the tissues improved and 5/6 of the tissues were not shaken apart after the shaking test. The thicknesses of the tissues stacked with the collagen adhesion treatment were measured, as shown in Figure 4b. Plane-shaped tissue with ca. 1.7 mm thickness can be fabricated by stacking five layers, which indicates the higher efficiency (i.e., to achieve the same thickness with the stacking of less tissues) of our proposed methods for fabricating thick tissues, in comparison to other methods such as the stacking of cell monolayers [22].

The maximum thickness achieved in our experiments is 4.1 mm, accomplished by stacking 19 layers of plane-shaped muscle tissues (Figure 4c). The red color of the tissue, inherited from the phenol red in the culture medium, gets denser with the increasing number of sheets, which also indicates the increased tissue thickness. The stacked tissue can be easily manipulated using tweezers, as shown in Figure 4d and Movie S1. Since the stacked tissues lack perusable channels within the tissues to provide sufficient nutrient delivery, the centimeter-order tissue blocks were not cultured after the stacking. In the future, methods for establishing such perusable channels, as well as perfusion culture protocols, are expected to further mature the tissue blocks.

## 4. Conclusions

In this work we present a cylindrical molding method for the biofabrication of plane-shaped skeletal muscle tissues, which can be further assembled to create subcentimeter-order tissue blocks. By molding the cell-laden hydrogel in a cylindrical cavity, the shrinkage of the hydrogel during tissue maturation can be controlled to create a uniformly shaped tissue. In comparison with other methods to control the tissue shrinkage, such as the methods using a pair of anchors to fix the tissue at two ends, our method can produce plane-shaped muscle tissues with uniform width and thickness. In experiments, we verified the uniformity of the fabricated tissue in terms of cell distribution and overall shape. Finally, we demonstrated the capability to fabricate subcentimeter-order tissue with a thickness of up to 4.1 mm, assembled by stacking 19 pieces of the muscle sheets. In the future, the fabrication process can be further scaled up by arraying the cylindrical tissue molds, integrating them with robotic systems for the automated dispensing of hydrogels, the plugging of the central pillars, as well as the cutting, expanding, and stacking of the plane-shaped skeletal muscle tissues into centimeter-order muscle tissue blocks.

## Data Availability

The data presented in this study are available on request from the corresponding author.

## Supplementary Materials

The following are available online at https://www.mdpi.com/article/10.3390/mi12111411/s1, Figure S1: photo image of the modified Petri dish for measuring the thickness of the tissue, Figure S2: the jig used for the stacking of the centimeter-order tissue block, Movie S1: a video showing the handling of the centimeter-order tissue block using a tweezer.
