# Supplementary material for: A Cylindrical Molding Method for the Biofabrication of Plane-Shaped Skeletal Muscle Tissue
Source: Micromachines (Basel). 2021 Nov 17;12(11):1411. doi: 10.3390/mi12111411 (PMC8625600; doi:10.3390/mi12111411)
Supplement: Supplementary file 1 [file micromachines-12-01411-s001.zip › Supplementary_Materials.pdf]

Supplementary Materials for

A cylindrical molding method for the biofabrication of wide-area skeletal muscle tissue with minimized material loss

Minghao Nie, Ai Shima, Kenta Fukushima, Yuya Morimoto, and Shoji Takeuchi

correspondence to: takeuchi@hybrid.t.u-tokyo.ac.jp

This PDF file includes:

Figure S1
Figure S2

Other Supplementary Materials for this manuscript includes the following:

Movie S1

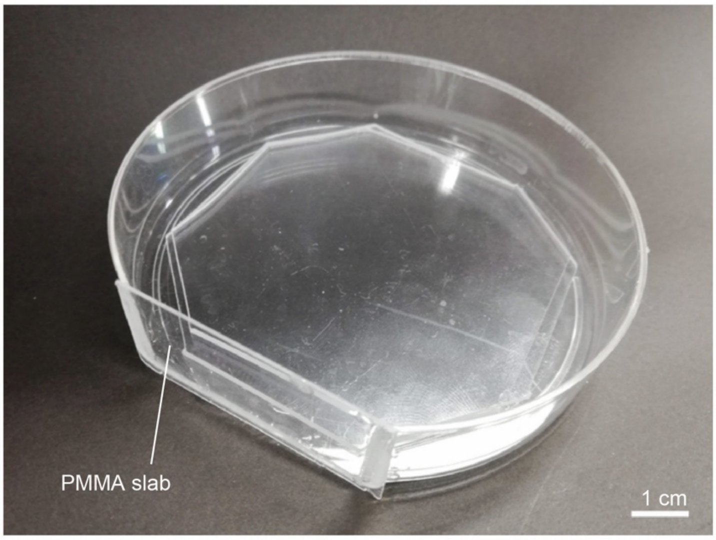

Figure S1.
Photo image of the modified petri dish for measuring the thickness of the tissue. Scale bar: 1 cm.

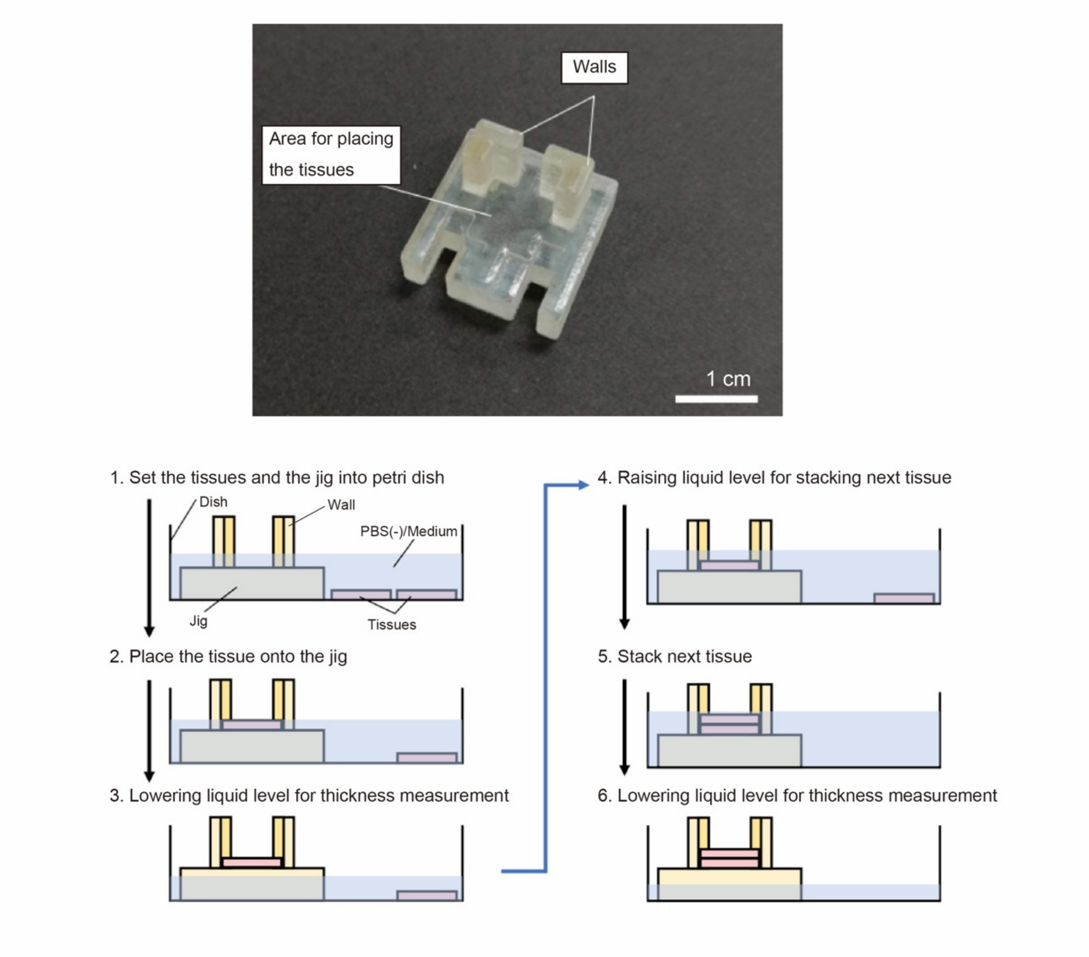

Figure S2.

The jig used for the stacking of the cm-order tissue block. Upper: photo image of the jig. Lower: illustrations depicting the steps for the stacking and thickness measurement of the tissues using the stacking of two tissue layers as an example. Scale bar: 1 cm.

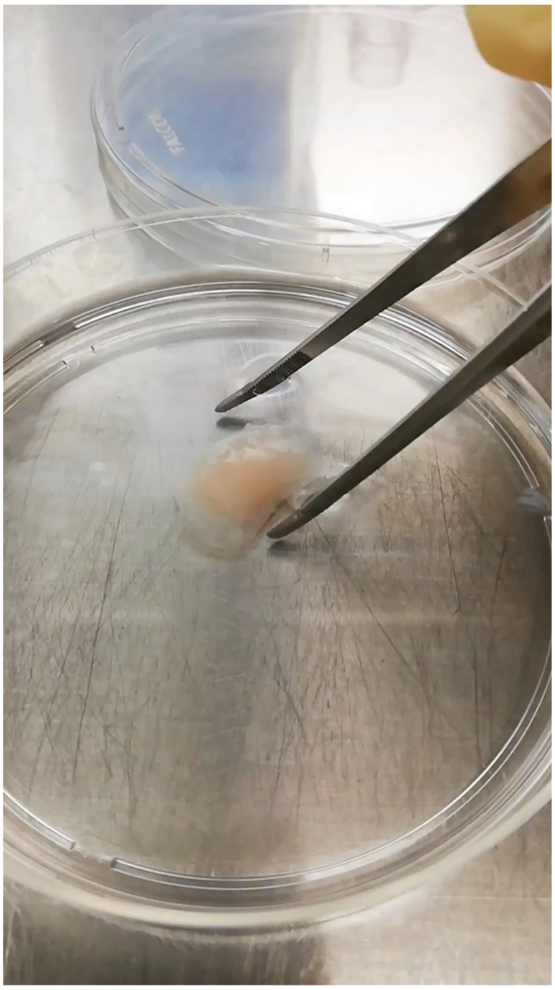

Movie S1

A video showing the handling of the cm-order tissue block using a tweezer.
